# Derivation of new pluripotent stem cells from human extended pluripotent stem cells with formative features and trophectoderm potential

**DOI:** 10.1111/cpr.13480

**Published:** 2023-04-13

**Authors:** Pinmou Zhu, Bohang Zhang, Ruiqi Sun, Jiachen Wang, Zhaode Liu, Xiaorui Liu, Min Yan, Yiqiang Cui, Jiahao Sha, Yan Yuan

**Affiliations:** ^1^ State Key Laboratory of Reproductive Medicine Nanjing Medical University Nanjing China; ^2^ State Key Laboratory of Reproductive Medicine Women's Hospital of Nanjing Medical University, Nanjing Maternity and Child Health Care Hospital, Nanjing Medical University Nanjing China

## Abstract

Previous studies have demonstrated the existence of intermediate stem cells, which have been successfully obtained from human naive pluripotent stem cells (PSCs) and peri‐implantation embryos. However, it is not known whether human extended pluripotent stem cells (hEPSCs) can be directly induced into intermediate stem cells. Moreover, the ability of extra‐embryonic lineage differentiation in intermediate stem cells has not been verified. In this issue, we transformed hEPSCs into a kind of novel intermediate pluripotent stem cell resembling embryonic days 8–9 (E8‐E9) epiblasts and proved its feature of formative epiblasts. We engineered hEPSCs from primed hPSCs under N2B27‐LCDM (N2B27 plus Lif, CHIR, DiH and MiH) conditions. Then, we added Activin A, FGF and XAV939 to modulate signalling pathways related to early humans' embryogenesis. We performed RNA‐seq and CUT&Tag analysis to compare with AF9‐hPSCs from different pluripotency stages of hPSCs. Trophectoderm (TE), primordial germ cells‐like cells (PGCLC) and endoderm, mesoderm, and neural ectoderm induction were conducted by specific small molecules and proteins. AF9‐hPSCs transcription resembled that of E8‐E9 peri‐implantation epiblasts. Signalling pathway responsiveness and histone methylation further revealed their formative pluripotency. Additionally, AF9‐hPSCs responded directly to primordial germ cells (PGCs) specification and three germ layer differentiation signals in vitro. Moreover, AF9‐hPSCs could differentiate into the TE lineage. Therefore, AF9‐hPSCs represented an E8‐E9 formative pluripotency state between naïve and primed pluripotency, opening new avenues for studying human pluripotency development during embryogenesis.

## Graphical abstract

We transformed primed‐derived hEPSCs into a new type of pluripotent stem cell AF9‐hPSC with similar transcriptomic properties to human E8‐E9 epiblasts by the addition of Activin A, bFGF and XAV939, which related to early human embryogenesis. AF9‐hPSCs possessed differentiation capacity towards three germ layer lineages and primordial germ cells‐like cells in vitro. In addition, AF9‐hPSCs allowed trophectoderm lineage induction.

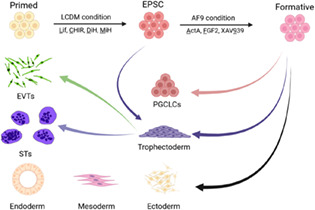

## 1 INTRODUCTION

Embryonic stem cells (ESCs) derive via pre‐implantation blastocyst inner cell mass (ICM). Both naïve and primed conditions for pluripotency have been investigated within murine ESCs and epiblast stem cells (mEpiSCs),^1^ which are transcriptionally analogous to embryonic E4.0‐E4.5 and E7.5 epiblasts, respectively.^2^, ^3^, ^4^ Similarly, human ESCs (hESCs) can also be divided into the naïve and primed pluripotent stages. However, conventional hESCs differ from their mouse counterparts and more closely resemble mEpiSCs in both transcriptome and methylation levels.^5^ Researchers have also found that conventional hESCs are closer to E14 post‐implantation human embryos at transcriptional and epigenetic levels and are termed primed hESCs. Subsequently, naïve hESCs have been successfully developed by various methods, and most exhibit the features of pre‐implantation epiblast‐stage embryos.^6^, ^7^, ^8^, ^9^

Recently, a new cell line with embryonic and extra‐embryonic potency in vivo has been established, which also known as the extended pluripotent stem cells (EPSCs).^10^, ^11^, ^12^ The most notable feature of EPSCs is that they can be chimeric and developing into both embryonic and extra‐embryonic tissues, with transcriptome properties similarly to between zygotes and the four‐cell (4C) stage.^10^, ^12^ Thus, human extended pluripotent stem cells (hEPSCs) have similar initial cell fate commitments to those seen in early pre‐implantation embryogenesis.

Despite these advances, the question of whether there is another pluripotency status naïve and primed pluripotency remains. Recent findings support a potential intermediary state occurring between such pluripotency status. Modulation of signalling pathways associated with peri‐implantation has resulted in the derivation of formative PSCs (fPSCs) in mice.^13–16^ Human XPSCs (hXPSCs) and formative pluripotent cells (hFSCs) have also been established through the transformation of human foreskin fibroblasts (HFFs), naïve hPSCs or human E5‐E6 embryos.^13^, ^14^ However, it is still not known whether intermediate stem cells could originate through hEPSCs. The elucidation of this issue will help to clarify whether hEPSCs are capable of crossing the naive state and transforming into intermediate stem cells, and thus discover the signalling pathways and molecular mechanisms that induce intermediate pluripotency.

In this study, we successfully transformed hEPSCs into an E8‐E9 epiblast‐like fPSCs (namely, AF9‐hPSCs) by modulation of FGF/Erk, TGFβ/Smad, together with WNT/β‐catenin signal transduction axis pathways, deemed as having crucial roles in human peri‐implantation embryo development. The AF9‐hPSCs showed self‐renewal and long‐term stability in vitro. Furthermore, we found that the cells corresponded to E8‐E9 in human embryonic epiblasts development and CUT&Tag sequencing confirmed the molecular features of human E8‐E9 epiblast pluripotency. Analysis of signalling pathways showed activation of the PI3K‐AKT, MEK and RAP1 pathways, known to be active during early human embryogenesis in AF9‐hPSCs. The AF9‐hPSCs also responded efficiently to three germ layer differentiation cues and could induce into primordial germ cells‐like cell (PGCLC) in vitro. In addition, the AF9‐hPSCs were found to be permissive for direct trophectoderm (TE) lineage induction.

## 2 MATERIALS AND METHODS

### 2.1 Ethics statement

Such an investigation was carried out with scrutiny and acceptance by Nanjing Medical University. All procedures had approval through Nanjing Medical University (2020027).

### 2.2 Cell culture

The hESC line H1 was a gift from Prof. Zhangjun. The hiPSC line DYR0100 iPSC (DYR0 iPSC) was procured through National Collection of Authenticated Cell Cultures. All cellular cultures were grown on Matrigel‐coated (Corning, 354,234) plates with E8 medium (Gibco, A1517001) and passaged every 4–6 days using ReleSR (Stem Cell, #05872). Cells were grown at 37°C, with 5% CO_2_ and 95% humidity. The medium was changed every day.

### 2.3 Conversion and growth of human EPSCs

Human EPSCs were induced from primed hPSCs. Specifically, conversion was performed 2 or 3 days following passage of the primed hPSCs. Mouse embryonic fibroblasts (MEFs) were inactivated with mitomycin C (Sigma, M4287) and seeded into the culture plates as feeder cells 24 h prior to the conversion. For the conversion, hPSC medium was exchanged using N2B27‐LCDM medium (10 ng/mL hLIF, 1 mM CHIR99021, 2 mM (S)‐(+)‐dimethindene maleate and 2 mM Minocycline hydrochloride) which was replaced each day. The growth of ESPCs was visible as dome‐shaped colonies.

### 2.4 Derivation of formative AF9‐hPSCs from hEPSCs

For conversion to AF9‐hPSCs, hEPSCs were treated with Accutase (Stem cell, #07920) and single‐cell suspensions were seeded on MEFs in N2B27‐LCDM and cultured for 1 day, whereby medium was exchanged using N2B27‐AF9 medium (N2B27 plus 20 ng/mL Activin A [A], 12 ng/mL bFGF [F] and 5 mM XAV939 [9]). The cells were cultured under normal conditions and the media were changed daily. Full conversion usually occurs within three to five passages. On passage, the cells were dissociated with Accutase and inoculated into fresh plates containing MEFs at split ratios between 1:10 and 1:15 every 3–4 days.

### 2.5 Chimeric experiments

AF9‐hPSCs were pre‐treated with 10 μM Y‐27623 for at least half an hour before injection. Around 5–8 singly dissociated cells were injected into mouse 8‐cell embryo. After microinjection, the embryo were cultured in CZB medium for 48 h. Fluorescent signals were observed using an inverted fluorescence microscope (Nikon) at 24 and 48 h post‐injection.

### 2.6 In vitro mesoderm, endoderm and neural induction

For mesoderm induction, AF9‐hPSCs were grown within 3 mM CHIR99021 and 500 nM LDN193189 (48 h), after which 20 ng/mL of bFGF was added from day 3 to 6.

For endoderm induction, AF9‐hPSCs were cultured within 100 ng/mL activin A, 100 nM PI‐103, 3 mM CHIR99021, 10 ng/mL bFGF, 3 ng/mL BMP4, together with 10 mg/mL heparin (24 h). This was followed by replacing through 100 ng/mL activin A, 100 nM PI‐103, 20 ng/mL bFGF, 250 nM LDN193189, together with 10 mg/mL heparin (additional 48 h culturing).

For neural induction, AF9‐hPSCs were cultured using 1 mM A83‐01 and 500 nM LDN193189 (6‐day treatment).

### 2.7 PGCLC induction

After dissociation of AF9‐hPSCs with Accutase, cells (6000 cells per well) were inoculated into low‐binding 96‐well plates and grown within GK15 (GMEM +15% Knockout Serum Replacement, 0.1 mM NEAA, 1 mM sodium pyruvate, 2 mM l‐glutamine and 0.1 mM 2‐mercaptoethanol) containing 500 ng/mL BMP4, 100 ng/mL hSCF, 1 mg/mL hLIF and 50 ng/mL EGF for 4 days. Rho‐linked kinase inhibitor Y‐27632 (10 mM) was added during the first day of induction.

### 2.8 Derivation of TS‐like cells

After dissociation with Accutase, single‐cell AF9‐hPSC suspensions were inoculated into fibronectin‐coated (10 ng/mL) plates and grown for 24 h, followed by replacing using hTSC medium (DMEM/F12 + 0.1 mM 2‐mercaptoethanol, 0.2% FBS, 0.3% BSA, 1% ITS‐X, 1.5 mg/mL l‐ascorbic acid, 50 ng/mL hEGF, 2 μM CHIR99021, 0.5 μM A8301, 1 μM SB431542, 0.8 mM VPA, together with 5 μM Y‐27632). The AF9‐TSCs were passaged using Accutase every 5–7 days, with 1:4 to 1:6 split ratios. AF9‐TSCs were routinely cultured at 37°C in hypoxic conditions (5% O_2_, 5% CO_2_).

### 2.9 Differentiation of STs and EVTs

AF9‐hPSCs were grown to ~70% confluence in TSC medium and dissociated with Accutase for 10 min at 37°C. For the induction of ST, AF9‐TSCs were seeded in a 6‐well plate pre‐coated with 2.5 μg/mL Col IV or 10 μg/mL fibronectin at a density of 1.5 × 10^5^ cells per well and cultured in 2 mL of ST medium (DMEM/F12 supplemented with 0.1 mM 2‐mercaptoethanol, 0.5% penicillin–streptomycin, 1% ITS‐X supplement, 4% knockout serum replacement, 0.3% BSA, 2.5 μM Y27‐632 and 2 μM forskolin. The medium was replaced at day 3, and the cells were analysed at day 6.

For EVTs induction, plates were pre‐coated with 1 μg/mL Col IV or Matrigel and cells were seeded in a 6‐well plate at a density of 1 × 10^5^ cells per well, and cultured in 2 mL of EVT medium (DMEM/F12 supplemented with 0.1 mM 2‐mercaptoethanol, 0.5% penicillin–streptomycin, 1% ITS‐X supplement, 4% KnockOut Serum Replacement, 0.3% BSA, 100 ng/mL NRG1, 7.5 μM A83‐01, 2.5 μM Y27‐632 and 2% Matrigel). After 3 days, the medium was changed to EVT medium with 0.5% Matrigel and without NRG1. Cells were collected until day 6.

### 2.10 Teratoma formation

Immunodeficient NOD‐SCID mice (6 weeks old) were injected subcutaneously with 1 × 10^7^ AF9‐hPSCs in 50 μL of DMEM‐Matrigel. The teratomas were harvested after 6–8 weeks and fixed in modified Davidson's fluid. The tissue was paraffin‐coated/sectioned/haematoxylin–eosin‐stained.

### 2.11 Assessment of clonal efficiency

After dissociation, the cells were sieved across a 40‐mm cell strainer, inoculated into 6‐well plates (1000 cells/well approx.). This was followed by culturing +/− 10 mM Y‐27632 with quantification of colonies (>20 cells) evaluated after 6 days. Clonal effectiveness was determined to be colony count divided using overall plated‐cell count.

### 2.12 Assessment of doubling time

After dissociation with Accutase, cells were counted and 1 × 10^5^ cells/well were inoculated into 24‐well plates harbouring feeder cells, cultured within AF9 medium. Growth rates were determined to be total cell number measured against time in culture.

### 2.13 Karyotype analysis

After incubation in KaryoMAX Solution (Gibco) for 1 h, cultures were placed again within suspension using 75 mM KCl and placed into incubation 37°C/30 min. Consequently, cells were fixed through 0.5 mL 3:1 (v/v) methanol:acetic acid KCl soln., followed by centrifugation (300 g/10 min). Supernatant was discarded, with cultures placed within incubation using 5 mL of cold methanol‐acetic acid fixative (30 min on ice). This was repeated before placing the cells on glass slides and staining via 300 nM 40‐6‐diamidino‐2‐phenylindole.

### 2.14 RNA extraction and qRT‐PCR (quantitative reverse transcription‐polymerase chain reaction)

Culture‐total RNA was collected through TRIzol. RNA was converted into cDNA using Hiscript III Reverse Transcriptase (Vazyme, R302‐01). qRT‐PCR was performed on a Q5 instrument (Applied Biosystems™) using AceQ qPCR SYBR Green Master MIX (Vazyme™, Q131‐02). GAPDH served as normalisation control‐gene. Datasets were assessed through delta–delta CT technique, with utilized primers illustrated within Table S1.

### 2.15 Immunofluorescence (IF)

At specified time points, cell cultures were 4% paraformaldehyde‐fixated (15 min/RT) followed by three 1 × PBS wash‐steps. Subsequently, cells were permeabilized for 120 min/RT within blocking solution (1 × PBS + 0.5% Triton X‐100/5% BSA). The blocking buffer was removed with no rinsing. Primary antibodies within blocking solution were then introduced/placed into incubation (overnight/4°C), with three subsequent 1 × PBS‐rinse‐steps. Fluorophore‐conjugated secondary antibodies in 3% BSA were then introduced/placed into incubation (RT/120 min/darkness). This was followed by four 1 × PBS‐wash‐steps within dark conditions. Carl Zeiss™ LSM8000® Confocal Microscopy imaging was consequently employed. Antibody specifications are listed hereunder:

Regarding human EPS cells; anti‐OCT4 (1:100, Santa Cruz™, sc‐5279); anti‐SOX2 (1:200, Abcam, ab92494); anti‐NANOG (1:200, CST, 4903S). The antibody used for formative AF9‐hPSCs was anti‐LEFTY1 (LifeSpan Biologicals, LS‐B5830). Anti‐TFAP2C (1:100, Santa Cruz, sc‐12,762), anti‐BLIMP1 (1:200, CST, 9115), and anti‐SOX17 (1:500, Neuromics, GT15094) were used for IF analysis of PGCLCs while for AF9‐induced TS‐like cells, the antibodies used were anti‐TFAP2C (1:100, Santa Cruz, sc‐12,762), anti‐CK7 (1:200, Zsbio, ZA‐0573) and anti‐GATA3 (1:200, R&D systems, AF2605).

### 2.16 Transcriptome Analysis

Total RNA was isolated using TRIzol. Sequencing was performed on an Illumina X Ten sequencer with a 150 bp paired‐end sequencing reaction. Raw Fastq files were trimmed by FASTp (v0.23.2),^17^ which removed adapters and low quality reads. The rRNA reads were removed using SortMeRNA software (v4.3.4).^18^ Hisat2 (v2.2.1) matched the trimmed reads to the human reference genome (GRCh38).^19^ Genes were quantified using the featureCounts function in the Rsamtools (v2.12.0) package. Normalized gene expression was expressed as counts per million mapped reads estimated by the edgeR (v3.38.1) package.^20^ Differential expression analysis was performed by the DEseq2 package version 1.36.0,^21^ and to explore the function of different groups of differentially expressed genes (DEGs), gene ontology (GO) and Kyoto Encyclopedia of Genes and Genomes (KEGG) pathway analysis was performed using the clusterProfiler package version 4.4.4.^22^ The Gene Set Enrichment Analysis (GSEA) analysis is based on the WikiPathway database through the function GSEA. Principal component analysis (PCA) was performed with the functions prcomp, and hclust in R. The visualisation results of RNA‐seq data analysis are presented by the ggplot2 package version 3.3.6 or the pheatmap package version 1.0.12.^23^

### 2.17 CUT&Tag sequencing

A CUT&Tag library was prepared through Hyperactive Universal CUT&Tag® Assay Kit for Illumina™ (Vazyme™, TD903). Specifically, Concanavalin A‐coated magnetic beads were rinsed in binding buffer while cells were collected, counted and rinsed in wash buffer. After immobilisation of the cells on the beads, buffer containing digitonin antibody was used for permeabilisation, with consequent incubating with primary antibodies for H3K4me3 (1:50, Abcam, 8580) and H3K27me3 (1:50, CST, 9733S) (overnight/4°C). Consequently, cultures were rinsed/exposed to second antibody (60 min/RT). Preparation of the pA‐Tn5‐adapter transposomes involved three washes (1 h each) of the pA‐Tn5 complex with Dig300 buffer. The pA‐Tn5 was then incubated with the cells in tagmentation buffer (60 min/37°C). After stopping tagmentation process, the DNA was solubilized by the addition of EDTA, SDS, together with proteinase K, with consequent extraction via phenol/chloroform/ethanol‐precipitation. DNA was finally amplified by PCR for insertion of the next‐generation sequencing indices and purified before sequencing the CUT&Tag libraries.

CUT&Tag raw files were quality controlled with FastQC v0.11.3, and human CUT&Tag reads were individually aligned to the human genome construct GRCh38 using bowtie2 v2.4.2 software.^24^ Genome browsing tracks for the UCSC Genome Browser were created using deepTools bamCoverage v3.5.1 (‐normalize Using Reads Per Kilobase Million [RPKM]).^25^ CUT&Tag peak distribution created using deepTools computeMatrix (−b 3000 ‐a 3000) and plotHeatmap.

## 3 RESULTS

### 3.1 In vitro deriving for intermediate pluripotent stem cells from human EPSCs

**FIGURE 1:**
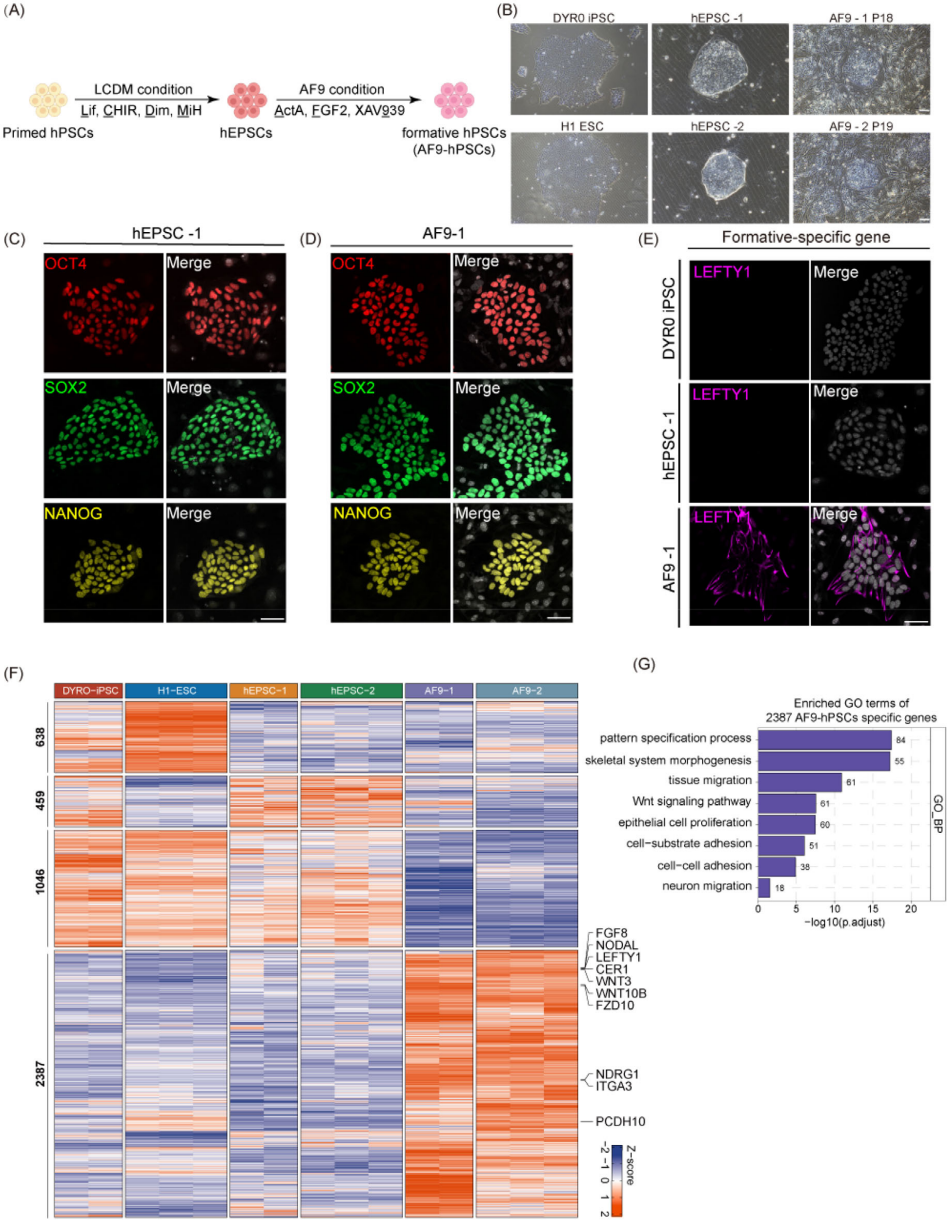
In vitro deriving for intermediate pluripotent stem cells from human EPSCs. (A) A schematic diagram showing AF9‐hPSCs generation from primed human PSCs. (B) Representative bright‐field images showing typical colony morphology of primed hPSCs, hEPSCs and formative AF9‐hPSCs. Scale bars indicate 100 μm. (C) Representative IF images showing that hEPSC‐1 expressed core pluripotency markers (OCT4, SOX2 and NANOG). Scale bars indicate 50 μm. (D) Representative IF images showing that AF9‐1 expressed core pluripotency markers (OCT4, SOX2 and NANOG). Scale bars indicate 50 μm. (E) Representative IF images showing the formative‐specific gene LEFTY1 was expressed in formative AF9‐1 PSCs but not expressed in primed DYR0 iPSCs and hEPSC‐1. Scale bars indicate 50 μm. (F) Heatmap clustering of differentially expressed genes in primed hPSCs (DYR0 iPSC and H1 ESC), hEPSCs (hEPSC‐1 and hEPSC‐2) and AF9‐hPSCs (AF9‐1 and AF9‐2). (G) GO analysis of 2387 specifically expressed genes of AF9‐hPSCs in (E). ESC, embryonic stem cells; EPSCs, extended pluripotent stem cells; GO, gene ontology; hEPSC‐1, human extended pluripotent stem cells‐1; IF, immunofluorescence.

Following a previous protocol,^10^ we induced human primed pluripotent stem cell DYR0 iPSCs and H1 ESCs into EPSCs, namely hEPSC‐1 and hEPSC‐2, respectively (Figure 1A). The obtained hEPSCs exhibited a typical dome‐shaped morphology (Figure 1B). IF showed that they expressed the core pluripotency markers OCT4, SOX2 and NANOG (Figure 1C and S1A). To further characterize hEPSCs, we conducted RNA‐seq and PCA showed that both hEPSC‐1 and hEPSC2 clustered in similar positions to those reported by Yang (Figure S1B). Consistently, in comparison with embryonic cells from pre‐implantation stages data,^26^ heatmap analysis based on highly variable features (HVFs) showed that hEPSC‐1, hEPSC2 and Yang's reported hEPSCs clustered together (Figure S1C).

To generate human intermediate PSCs from hEPSCs, we treated the cells with FTW medium containing FGF (F), the TGF/β agonist Activin A (T), and a WNT agonist, CHIR99021 (W).^14^ The hEPSCs were seeded on mitomycin C‐treated MEFs, changing the medium to FTW medium the following day. This led to the attachment and outgrowth of most of the hEPSC clones, followed by either differentiation or death after passage. As WNT signalling is known to induce mesodermal differentiation of hPSCs and initiate primitive streak formation,^27^, ^28^ we replaced the WNT agonist CHIR99021 with the WNT inhibitor XAV939 (namely, the AF9 condition) (Figure 1A). After about 6–7 days of induction, we found that the original dome‐shaped morphology of the hEPSCs (hEPSC‐1 and hEPSC‐2) became slightly flattened with well‐defined intercellular boundaries (Figure 1B). IF staining showed that while these transformed cells still expressed OCT4, SOX2 and NANOG, they had also begun to express the formative‐specific gene LEFTY1 (Figure 1D,E and S2A,B). In addition, AF9‐hPSCs exhibited alkaline phosphatase activity (Figure S2C). Thus, FGF and TGF‐β/Smad signal triggering together with inhibition of the WNT/β‐catenin pathway were essential for stabilizing hEPSC‐1 and ‐2‐derived PSCs, hereafter referred to as AF9‐1 and AF9‐2, respectively.

We then evaluated the functional pluripotency of the AF9‐hPSCs using in vivo differentiation of teratomas. This showed differentiation of the AF9‐hPSCs into cells from all three germ lineages (Figure S2D). Furthermore, the cells remained stable in terms of both growth and karyotype after prolonged culture (Figure S2E,F). In addition, AF9‐hPSCs were dependent on the treatment of ROCK inhibitor when passaged (Figure S2G). To evaluate chimeric capability of AF9‐hPSCs, we injected DsRed‐labelled AF9‐hPSCs into mouse 8‐cell embryo and cultured them in vitro with CZB medium. Two days later, AF9‐hPSCs were incorporated in 25 out of 69 (36%) ICMs and expressed OCT4 co‐staining with human‐specific nuclear matrix antigen (Figure S2H,I and Table S2). These findings showed that AF9‐hPSCs were capable of contributing to chimera formation in mouse embryos.

To assess whether the AF9‐hPSCs harboured intermediate pluripotency features, we performed RNA‐seq and analysed the differential expressed genes (DEGs) among the primed hPSCs, hEPSCs, and AF9‐hPSCs (Figure 1F). We identified 2397 genes specifically upregulated in AF9‐hPSCs. GO enrichment showed that the AF9‐hPSC‐specific genes were mainly associated with cellular response to tissue migration, cell fate commitment, ossification, and so on (Figure 1G). Taken together, these findings indicated that AF9‐hPSCs belong to a pluripotent stage distinct from either the naïve or primed pluripotent conditions.

### 3.2 AF9‐hPSCs harbour intermediate pluripotency features

**FIGURE 2:**
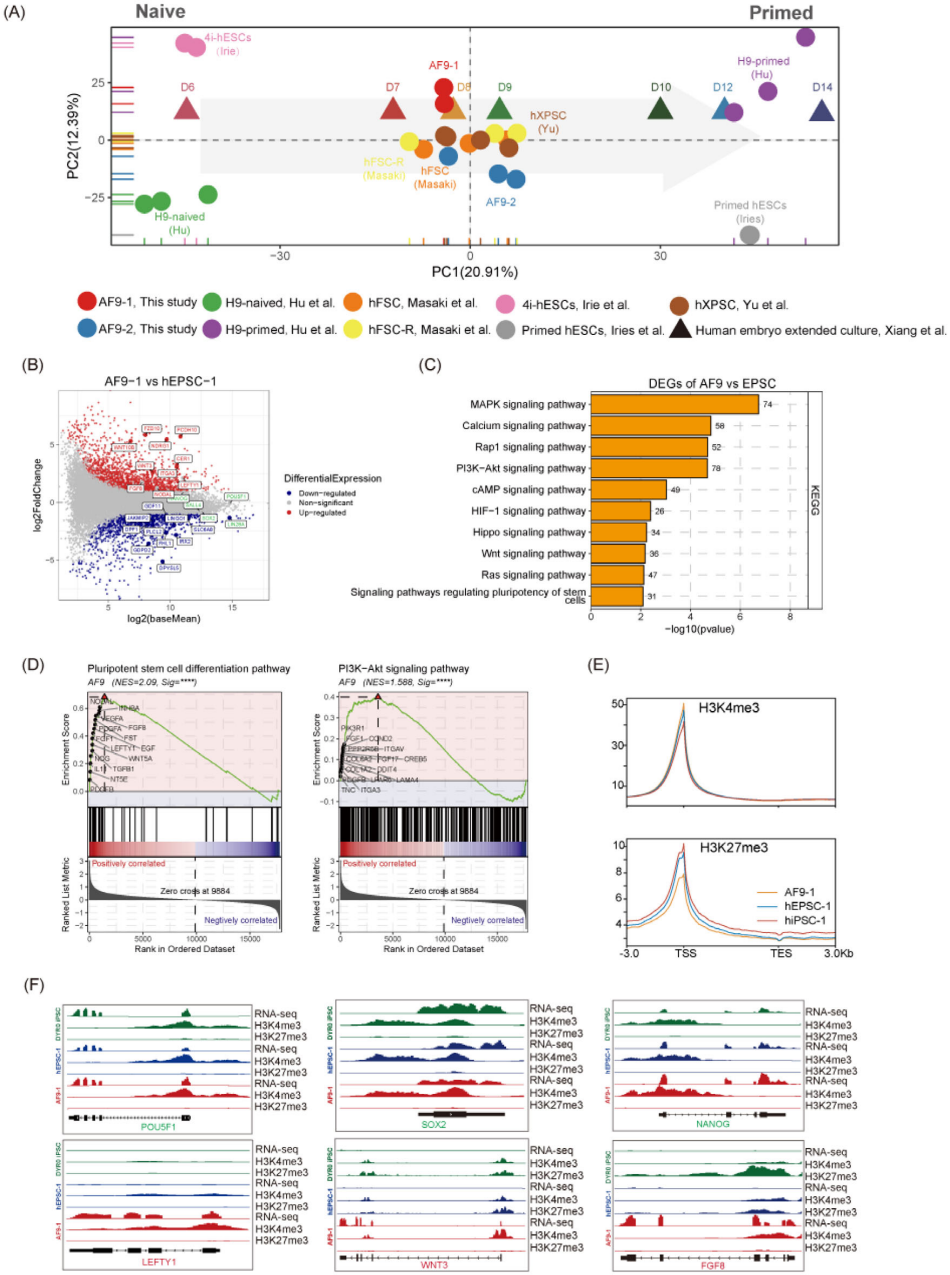
AF9‐hPSCs harbour intermediate pluripotency features. (A) A PCA plot of RNA‐seq data from in vitro (4i hESCs, H9 naïve and primed ESCs, hXPSCs, hFSCs and AF9‐hPSCs) and in vitro (human E6, E8, E10, E12 and E14 epiblast) samples from Xiang et al (2021). (B) Matrix antigen plot showing mean Fragments per Kilobase Million (FPKM) value against fold change per gene in AF9‐1 versus hEPSC‐1. Gene symbols are shown for selected formative (red), EPS (blue) and pluripotency (green) genes. (C) Kyoto encyclopedia of genes and genomes pathway analysis in AF9‐hPSCs compared to hEPSCs. (D) GSEA pathway analysis in AF9‐hPSCs compared to hEPSCs based on the WikiPathways database. (E) Average H3K4me3 and H3K27me3 signalling in all RefSeq genes in AF9‐hPSCs (AF9‐1), hEPSCs (hEPSC‐1) and primed hPSCs (DYR0 iPSC), represented as normalized RPKM values. (F) RNA‐seq, H3K4me3 and H3K27me3 tracks of selected pluripotency and formative genes in DYR0 iPSC, hEPSC‐1 and AF9‐1. ESC, embryonic stem cells; EPSCs, extended pluripotent stem cells; GO, gene ontology; hEPSC‐1, human extended pluripotent stem cells‐1; IF, immunofluorescence.

To further confirm the pluripotency state of AF9‐hPSCs, we compared RNA‐seq analysis of both AF9‐hPSCs with published human epiblasts from cultured embryos,^26^, ^29^ as well as naïve hESCs (4i‐hESCs)^30^ and primed hESCs datasets.^30^ PCA indicated the close proximity of the AF9‐hPSCs with separation from naive and primed PSCs (Figure 2A and S3A). Furthermore, the AF9‐hPSCs, naïve hPSCs, and primed hPSCs clustered close to epiblasts from human embryonic stages E8‐E9, E6 and E12‐E14, respectively (Figure 2A), indicating that AF9‐hPSCs represented an intermediate state clearly separated from both human naïve and primed pluripotency. In addition, upregulation of genes associated with formative cells was observed while EPS‐associated genes were downregulated in AF9‐hPSCs compared with hEPSCs (Figure 2B and S3B).

To better investigate the characteristics and differences among previous reported hXPSCs, hFSCs and AF9‐hPSCs, we compared their transcriptomes, finding that AF9‐hPSCs clustered with hXPSCs and hFSCs (Figure 2A). Additionally, heatmap of their DEGs was divided into four clusters (Figure S3C). Genes upregulated in AF9‐hPSCs and hXPSC were cluster 1, and enriched GO terms of which were mainly concentrated on matrix organisation, gastrulation, etc. Besides, genes upregulated in AF9‐hPSCs and hFSCs were cluster 3, and enriched GO terms were related to regulation of membrane potential, cell–cell adhesion, etc. (Figure S3D). These enriched functions were very closely associated with embryonic peri‐implantation development, indicating that AF9‐hPSCs may reflect the features of peri‐implantation embryos.

To further identify the signalling pathways involved in the induction of AF9‐hPSCs, we conducted KEGG analysis of genes from the RNA‐seq datasets and found that AF9‐hPSCs were enriched in pathways related to the PI3K‐AKT, MAPK and RAP1 signalling pathways, among others (Figure 2C). Genes associated with these signalling pathways that were significantly enriched and appeared frequently were FGF2, EGF and FGFR (Figure S4A). Notably, several recent studies have shown that the above signalling pathways played critical roles during pre‐implantation embryonic development, embryonic renal differentiation, TE development, invasion and migration.^31^, ^32^, ^33^ Moreover, using the GSEA Kiwi pathway analysis, we found that the enriched signalling pathways in AF9‐hPSCs were mainly associated with the pluripotent stem cell differentiation pathway, PI3K‐AKT signalling pathway, and embryonic stem cell pluripotency pathways, all of which are strongly correlated with embryonic development during implantation (Figure 2D and S4B,C). We also performed CUT&Tag analysis and found that AF9‐1 showed the highest level of H3K4me3 and the lowest H3K27me3 levels around the TSS region compared to DYR0 iPSCs and hEPSC‐1 (Figure 2E and S5A). Compared to hEPSC‐1 and DYR0‐iPSC, 785 H3K4me3‐high genes and 4011 H3K27me3‐low genes were identified in AF9‐1. GO analysis revealed that H3K4me3‐high genes were mainly enriched in pattern specification process and cell fate commitment, etc. In the meanwhile, genes enriched in H3K27me3‐low genes included histone modification and stem cell population maintenance, etc. These pathway results are consistent with previous GO results for gene activation at the RNA level in AF9‐1 (Figure S5B). Enrichment of H3K4me3 modifications together with reduced H3K27me3 modification levels were identified inside promoter regions for a selection of formative and pluripotency‐associated genes, such as LEFTY1, WNT3 and FGF8, in AF9‐1, which correlated with RNA expression (Figure 2F).

Together, these results indicated that AF9‐hPSCs corresponded to a formative stem cell identity and thus reflected the properties of human peri‐implantation embryos. In summary, these results suggest that the AF9‐hPSCs were novel formative stem cells with E8‐E9 epiblast properties between naive and primed pluripotency.

### 3.3 AF9‐hPSCs exhibit PGC and three germ layers lineage competence

**FIGURE 3:**
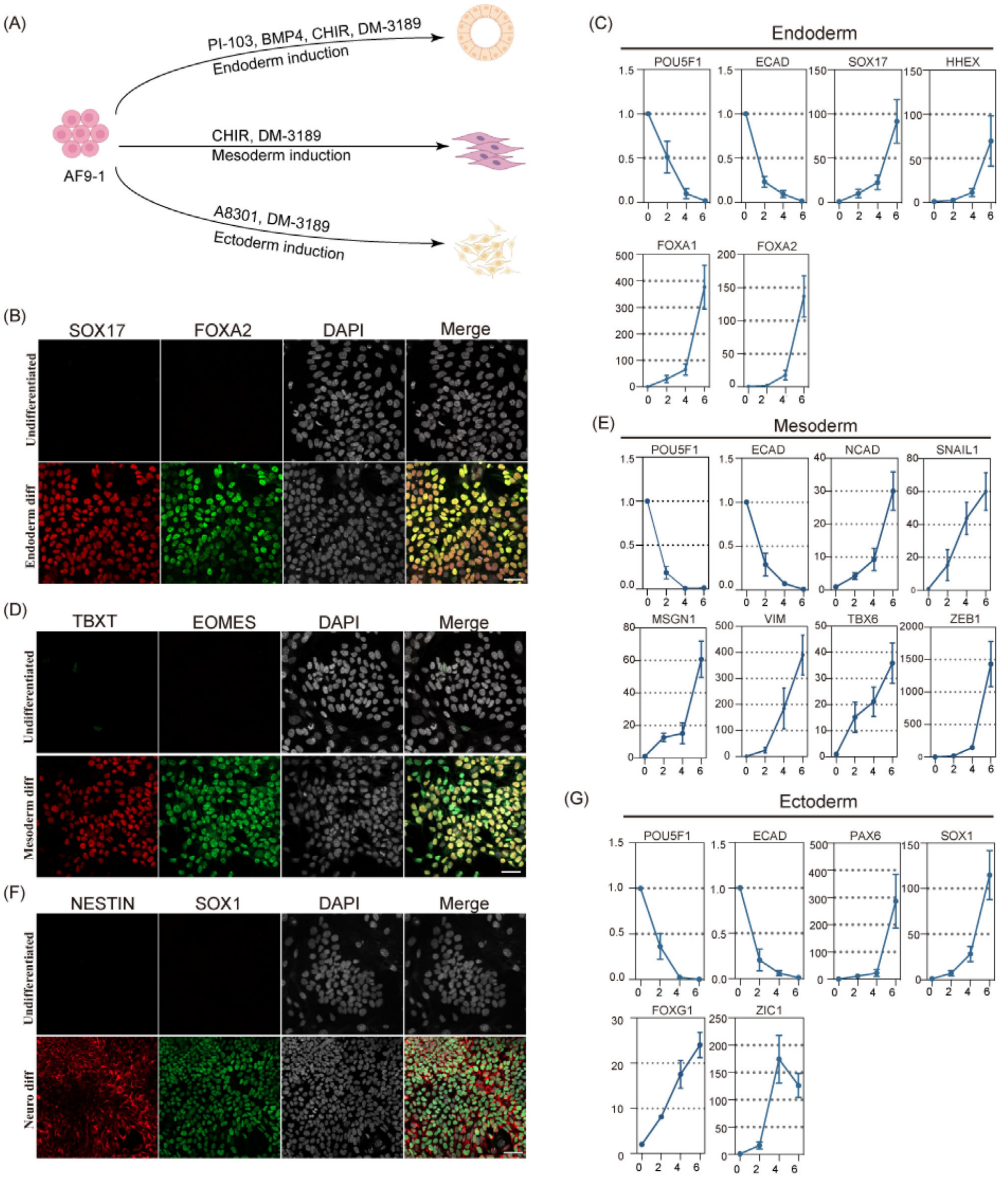
AF9‐hPSCs respond productively to lineage induction cues in vitro. (A) A schematic diagram showing in vitro endoderm, mesoderm and ectoderm induction from AF9‐hPSCs. (B) SOX17 and FOXA2 immunostaining of AF9‐hPSCs after endoderm induction. Scale bar, 50 μm. (C) qRT‐PCR analysis of AF9‐hPSCs differentiated into endoderm for 6 days. Error bars represent SD from technical triplicates. (D) TBXT and EOMES immunostaining of AF9‐hPSCs after mesoderm induction. Scale bar, 50 μm. (E) qRT‐PCR analysis of AF9‐hPSCs differentiated into mesoderm for 6 days. Error bars represent SD from technical triplicates. (F) NESTIN and SOX1 immunostaining of AF9‐hPSCs after neural ectoderm induction. Scale bar, 50 μm. (G) qRT‐PCR analysis of AF9‐hPSCs differentiated into neural ectoderm for 6 days. Error bars represent SD from technical triplicates. qRT‐PCR, quantitative reverse transcription‐polymerase chain reaction.

Current theory is that since naïve hPSCs correspond to the pre‐implantation embryonic state, they cannot be directly induced to somatic lineages but must pass through an intermediate pluripotency stage.^34^ To investigate whether the AF9‐hPSCs could undergo direct differentiation to the three germ layers, we applied established methods of lineage induction (Figure 3A).^35^, ^36^, ^37^ After endoderm induction, the formation of FOXA2‐ and SOX17‐positive cells were detected by IF (Figure 3B). In addition, qPCR demonstrated the downregulation of pluripotency‐related markers (POU5F1 and ECAD) and upregulation of endoderm‐related markers (SOX17, HHEX, FOXA1 and FOXA2; Figure 3C). Similar effects were observed after inducing mesoderm differentiation, with increased expression of TBXT and EOMES in cells and the upregulation of TBX6, VIM and MSGN1 together with markers of the epithelial‐mesenchymal transition such as NCAD, SNAIL1 and ZEB1 (Figure 3D,E). Furthermore, we initiated differentiation of AF9‐hPSCs to the neural ectoderm lineage by inhibiting SMAD signalling, resulting in increased numbers of NESTIN and SOX1 double positive cells (Figure 3F) as well as increased levels of neural epithelial markers (ZIC1 and PAX6) and anterior central nervous system markers (FOXG1) (Figure 3G), indicating the acquisition of neural lineage developmental competence.

**FIGURE 4:**
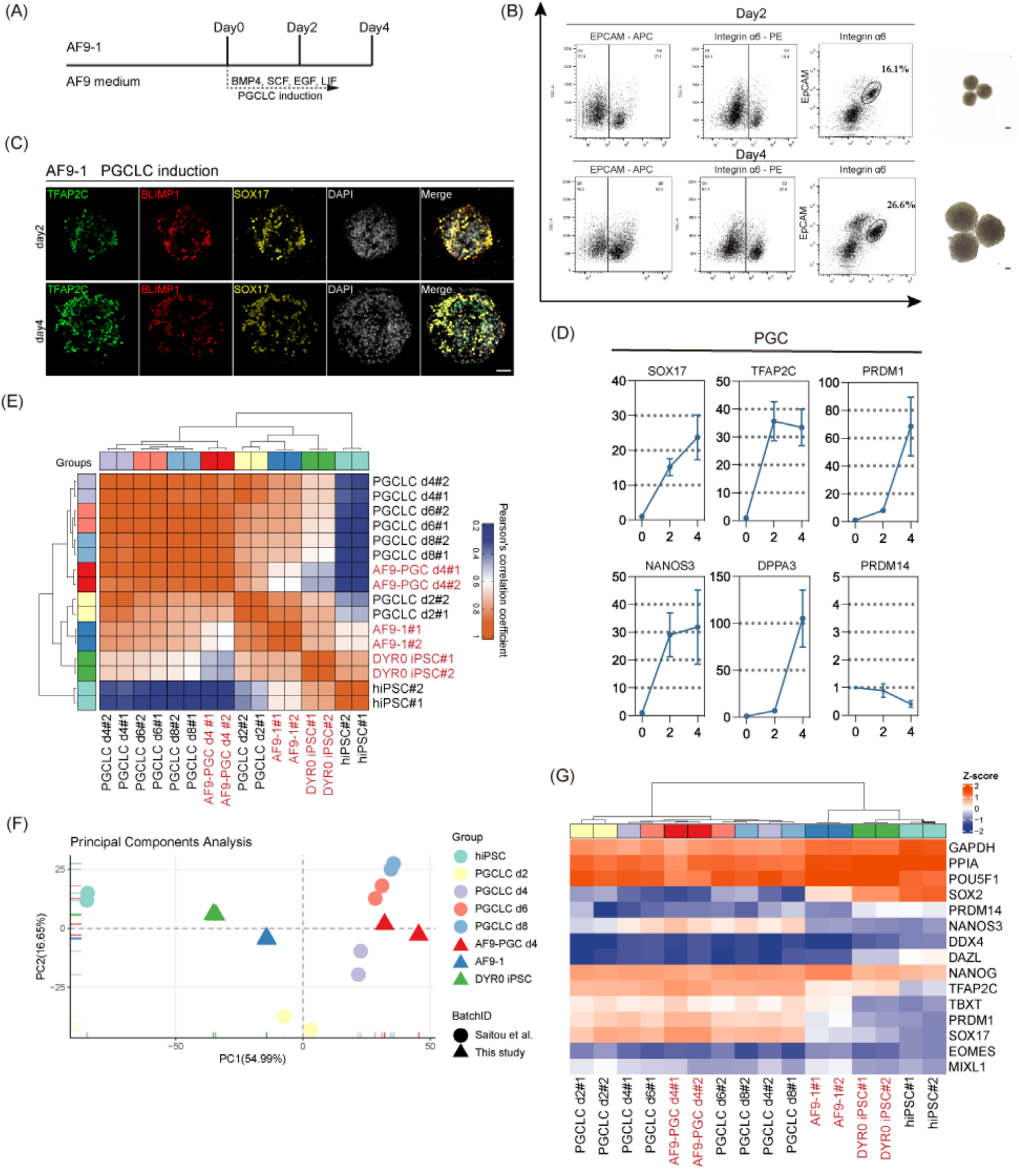
AF9‐hPSCs exhibit PGC competence. (A) A scheme for the PGCLC induction from AF9‐hPSCs. (B) Typical examples for the bright‐field images and FACS analysis during PGCLC induction from day 2 to day 4. Scale bars, 100 μm. (C) Representative IF (SOX17, TFAP2C and BLIMP1) images of PGCLC induction from AF9‐hPSCs. Scale bars, 50 μm. (D) Gene expression dynamics during PGCLC induction from AF9‐hPSCs (mean ± SD; *n* = 3, biological replicates). (E) Heatmap of correlation coefficients of the gene‐expression profiles among the indicated cells. AF9‐1, AF9‐hPSCs induced by hEPSC‐1; AF9‐PGC d4, 4 days after PGCLC induction of AF9‐1; hiPSC, established by Sasaki et al.^38^; PGCLC d4, d6, d8, 4, 6, 8 days PGCLC induction of hiPSCs by Sasaki et al.^38^ (F) PCA of the indicated cell types during PGCLC induction. The cells as annotated are plotted in two‐dimensional spaces defined by PC1 and 2. (G) Heatmap of the relative expression of selected genes associated with DYR0 iPSC, AF9‐1, AF9‐PGC d4, hiPSC, PGCLC d2, d4, d6 and d8. PGCLC, primordial germ‐cells like cells.

To better understand the differentiation capabilities of AF9‐hPSCs, we conducted in vitro induction of PGCLCs using established methods^38^ (Figure 4A). Two PGCLC‐specific cell surface markers, integrin α6 and EpCAM, were used for selective labelling of the cells during purification. BMP4 was used for the induction, and it was observed by day 2 of induction 16.1% of the cells were integrin α6/EpCAM+, increasing to 26.6% by day 4 (Figure 4B). As shown by IF, the cells expressed TFAP2C, PRDM1 and SOX17 (Figure 4C). We performed qPCR analysis and found that the integrin α6/EpCAM+ cells showed significantly increased levels of early PGC markers, including SOX17, TFAP2C, PRDM1, NANOS3 and DPPA3, compared to AF9‐hPSCs (Figure 4D). Similarly, the Pearson's correlation coefficient heatmap indicated that AF9‐PGCLCs on day 4 clustered with primed hPSCs‐induced day 6 PGCLCs (Figure 4E). Consistently, PCA revealed that day 4 AF9‐PGCLCs were distinct from AF9‐hPSCs and primed PSCs but clustered close to reported primed hPSC‐induced PGCLCs (Figure 4F). In addition, we found that both day 4 AF9‐PGCLCs and reported primed hPSC‐induced PGCLCs on days 2, 4, 6 and 8 expressed high levels of early PGC markers (Figure 4G). These findings indicated that AF9‐hPSCs showed the characteristics of both molecular and cellular pluripotency and were capable of lineage differentiation and PGCLC induction in vitro.

### 3.4 AF9‐hPSCs possess TE lineage potential

**FIGURE 5:**
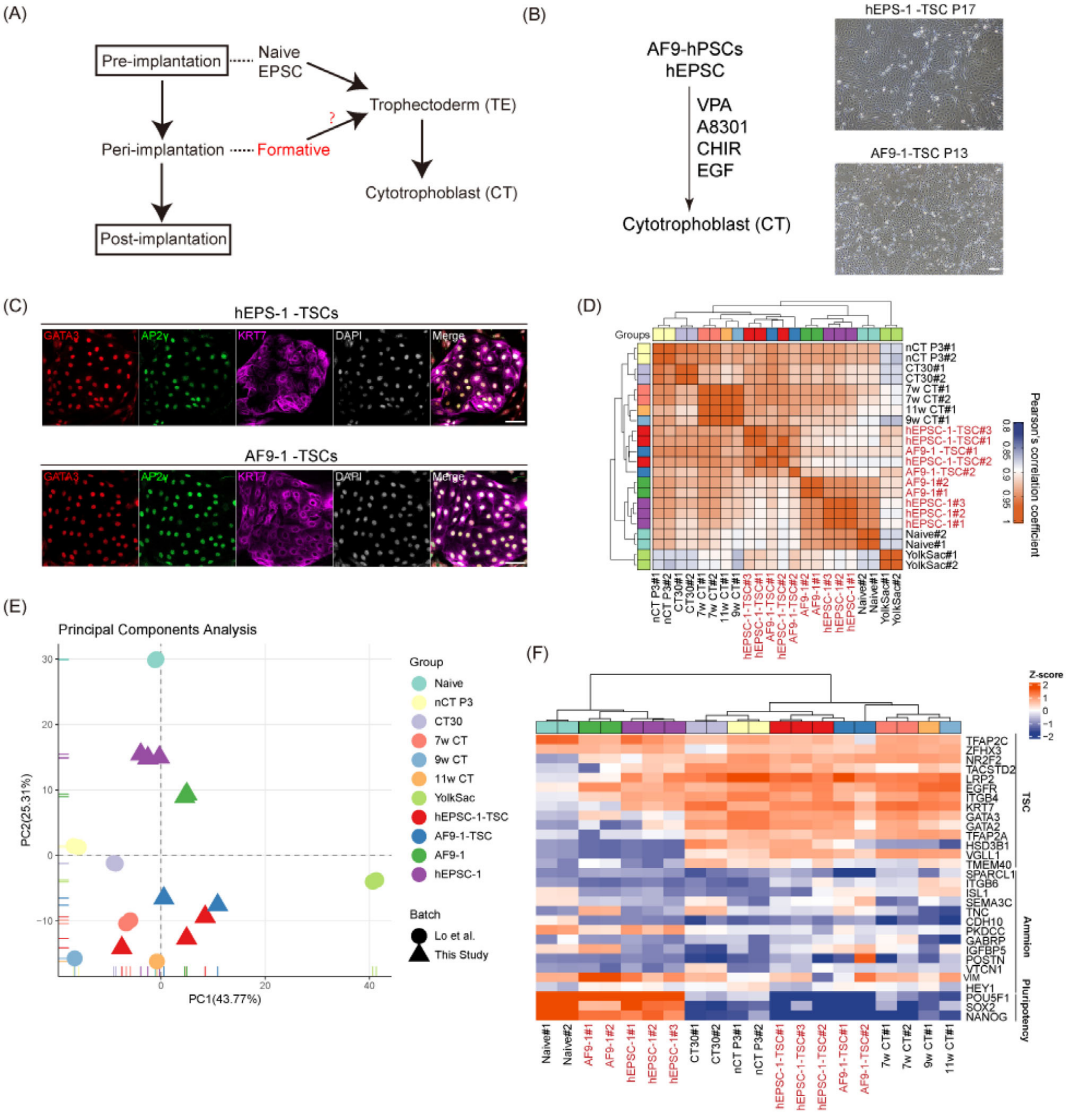
AF9‐hPSCs possess trophectoderm (TE) lineage potential. (A) Schematic of human trophoblast development. TE in pre‐implantation embryos differentiates into cytotrophoblasts (CT) during implantation. (B) A scheme for the trophoblast stem cell (TSC) induction from AF9‐hPSCs and representative bright‐field images showing typical colony morphology of extended pluripotent stem (EPS) cells derived TSCs and AF9‐hPSCs derived TSCs. Scale bar, 100 μm. (C) Representative images of EPS‐TSC and AF9‐TSC, detecting the expressing TE lineage‐specific markers (GATA3, TFAP2C and CK7). Scale bar, 50 μm. (D) Heatmap of correlation coefficients of the gene‐expression profiles among the indicated cells. Naïve, naïve PSCs; hEPSC‐1,‐2, hEPSC induced by DYR0 iPSC and H1 ESC, respectively; AF9‐1, ‐2, AF9‐hPSCs induced by hEPSC‐1 and hEPSC‐2, receptively; AF9‐1‐TSC, TSC induced by AF9‐1; hEPSC‐1‐TSC, TSC induced by hEPSC‐1; 7 w, 9 w and 11 w CT, TACSTD+ENPEP+SIGLEC6+ placental chorionic villi at 7, 9 and 11 gestational weeks reported by Io et al.,^39^ respectively; CT30, placenta‐derived TS cells established by Okae et al.^43^ nCT, naive PSC‐derived TE differentiates into CTs reported by Io et al.^39^ Yolk sac samples reported by Cindrova‐Davies et al.^44^ (E) PCA of indicated cell types in (D). (F) Heatmap of the relative expression of selected genes associated with cell types in (D).

The formation of TE is an important step during embryogenesis. Recently, various approaches have been attempted to induce TE from naïve hPSCs and hEPSCs and establish culture methods to proliferate trophoblast stem cells (TSCs) in vitro.^39^, ^40^, ^41^ To determine whether formative AF9‐hPSCs can be transformed into TSCs (Figure 5A), we changed the medium from the initial AF9 medium to medium promoting TSC induction together with culture on MEFs. This led to the appearance of colonies with TSC morphology after 5–7 days (Figure 5B). The colonies were selected and cultured under hTSC conditions, leading to the establishment of stable cell lines. Similar to hEPSC‐induced TSCs (hEPSC‐1‐TSCs), AF9‐induced TSCs (AF9‐1‐TSCs) showed typical TSC morphological features and expressed TSC markers (GATA3, TFAP2C and CK7; Figure 5C). In addition, to confirm that indeed AF9‐hPSCs and not residual EPSCs can be induced into extraembryonic lineages, we planted AF9‐hPSCs single cells on 96‐well plates to ensure that the clones grown in each well were uniform. Then we randomly picked 10 clones and tried to induce them into TSCs. The results showed that all 10 clones were successfully induced as TSCs and immunofluorescence staining confirmed its identity (Figure S6). Consistently, the Pearson's correlation coefficient heatmap indicated that the AF9‐1‐TSCs, hEPSC‐1‐TSCs and 7, 9 and 11 w CTs have more similar gene expression profiles than hEPSCs, naïve hPSCs and AF9‐hPSCs (Figure 5D). PCA also revealed that AF9‐1‐TSCs and hEPSC‐1‐TSCs clustered close to human embryonic 7, 9 and 11 w CTs (Figure 5E). Moreover, we found that AF9‐1‐TSCs, hEPSC‐1‐TSCs and 7, 9, and 11 w CTs expressed TSC‐related markers but did not express either amnion or pluripotency markers (Figure 5F). These results showed that AF9‐hPSCs can differentiate into the TE lineage. In vivo, CTs can further develop into syncytiotrophoblast (STs) and extravillous trophoblast (EVTs). To identify the differentiation potential of AF9‐TSCs, we induced AF9‐TSCs according to previously reported induction methods^42^, ^43^ (Figure S7A). When forskolin was added, the morphology of AF9‐TSCs transformed into multinucleated syncytia (Figure S7B). Immunofluorescent staining showed that the cells began to express ST marker human gonadotropin (hCG‐β; Figure S7D). We also performed ELISA assays and found that AF9‐STs secreted high concentrations of hCG‐β compared with AF9‐TSCs (Figure S7C). The above results indicated that we successfully induced AF9‐TSCs into STs. In addition, we performed induction of EVTs. After 6 days of induction with NRG1 and matrigel, we found that the cells became a spindle‐like morphology (Figure S7B), and the expression of HLA‐G was detected (Figure S7E). These data showed that AF9‐hPSCs possessed the full inducibility of TSCs, STs and EVTs, implying that human pluripotent stem cells in intermediate pluripotency have not lost their TE differentiation potential.

## 4 DISCUSSION

Increasing evidence has demonstrated that PSCs can capture and reflect the embryonic developmental state in vitro. Recently, several investigations confirmed existence for the intermediate pluripotency stage in both humans and mice.^13^, ^14^, ^15^, ^16^ Furthermore, through the use of similar culture conditions, hXPSCs and hFSCs could be successfully established from HFF, naïve hPSCs, or human embryos.^13^, ^14^ However, it is still unclear whether formative PSCs can be obtained directly from hEPSCs. Here, we derived a novel formative stem cell AF9‐hPSC line with similar transcriptomic properties to human E8‐E9 epiblasts. We further demonstrated that the AF9‐hPSCs possessed differentiation capacity towards all such germ‐layer lineages and PGCLCs. In addition, AF9‐hPSCs allowed extra‐embryonic lineage induction. These results help to explain the interconversion relationships between epiblasts in different pluripotency states during early human embryonic development and discover the signalling pathways and molecular mechanisms that induce and maintain intermediate pluripotency.

In comparison with previously reported hXPSCs and hFSCs, AF9‐hPSCs were derived from hEPSCs and maintained their E8‐E9 epiblast properties through inhibiting WNT/β‐catenin signalling rather than its activation as was shown with hXPSCs.^14^ This suggests WNT/β‐catenin axis could not always be activated or inhibited during the development of peri‐implantation embryos, implying the presence of different types of intermediate phases during early human embryogenesis. Furthermore, KEGG and GSEA pathway analyses indicated that embryo‐related signalling pathways, such as PI3K‐AKT, MAPK, and RAP1 were strongly activated, and previous reports have pointed out that these pathways are especially associated with pre/peri‐implantation embryogenesis, embryonic renal differentiation, and TE development.^31^, ^32^, ^45^ Therefore, such transcriptome and pathway analysis of AF9‐hPSCs indicated their formative pluripotency identity and thus may represent an alternative state of peri‐implantation pluripotency in humans.

The ability of mouse ESCs, mouse epiblast stem cells (mouse EpiSCs), naïve hPSCs and primed hPSCs to be induced to PGCLCs has been reported.^30^, ^38^, ^46^, ^47^, ^48^ This investigation identified formative AF9‐hPSCs responding directly following BMP4 stimulation, differentiating towards PGCLCs in vitro with high efficiency. PCA also showed the transcriptomic similarities between AF9‐induced PGCLCs and primed hPSC‐induced PGCLCs. Additionally, the highly successful induction of endoderm, mesoderm and ectoderm confirmed that the AF9‐hPSCs possessed efficient lineage differentiation capabilities. Therefore, the AF9‐hPSCs were highly capable of induction to PGCLCs and all three germ layers, also explaining their formative identity.

More importantly, we also found that the AF9‐hPSCs possessed TE lineage potential. Smith et al. proposed a pluripotent lineage progression model in which there is retention of human TE potency until the intermediate transition to amnion epiblast competence.^41^ Here, we found that when exposed to hTSC culture conditions, AF9‐hPSCs can transform to TSCs, showing the typical morphology of primary TSCs, while expressing TE but not amnion markers, and transcriptional similarity to EPS‐derived TSCs and 7, 9 and 11 w CTs. These findings suggest that AF9‐hPSCs possess the intrinsic potential for TE formation. Furthermore, AF9‐TSCs can differentiate into STs and EVTs, which confirmed the potential for AF9‐hPSCs to form functional cell types of trophoblasts. We speculate that this retention of this capacity may be more widespread in human pre/peri‐implantation embryos than previously thought.

Overall, AF9‐hPSCs are likely to be highly useful for augmenting our understanding of mammalian pluripotency. To date, arguments for peri‐implantation pluripotency have focused more on the mechanisms that confer multi‐lineage competence. During embryonic development, cells undergo a highly efficient and precise process of differentiation governed by strict developmental rules. However, in a culture environment, the transition from pluripotency may become disrupted due to differences between the internal and external conditions, leading to potentially severe consequences. Thus, the clarification of the programmed transitions in relation to pluripotency is necessary for understanding how lineages are decided and enabled. Furthermore, recapitulating the transition from pre‐implantation blastocysts to peri‐implantation embryos in vitro may enhance the regulation of quality of pluripotent stem cell differentiation. The findings of this study may thus provide useful information for biotechnology, regenerative medicine and reproductive biology.

## AUTHOR CONTRIBUTIONS

Pinmou Zhu performed the majority of the experiments, interpreted the data and wrote the manuscript. Bohang Zhang and Jiachen Wang analysed and interpreted data. Ruiqi Sun, Min Yan and Xiaorui Liu performed the majority of the experiments related to animal experiments. Yiqiang Cui and Zhaode Liu performed CUT&Tag. Yan Yuan and Jiahao Sha provided direction in experimental design and supervised the project.

## FUNDING INFORMATION

This study was supported by National Natural Science Foundation of China grant 92068109 and 82122025; National Key R&D Program grant 2021YFC2700302 and 2021YFC2700200, Natural Science Foundation of the Jiangsu Higher Education Institutions of China 21KJA310007 and Science Foundation of Gusu School GSKY20220101. The authors especially thank Biorender® for the cartography.

## CONFLICT OF INTEREST STATEMENT

The authors declare that the research was conducted in the absence of any commercial or financial relationships that could be construed as a potential conflict of interest.

## Supporting information


**Data S1.** Supporting information

## Data Availability

The data that support the findings of this study are available within the manuscript and supplementary materials.
