# Supplementary material for: Derivation of new pluripotent stem cells from human extended pluripotent stem cells with formative features and trophectoderm potential
Source: Cell Prolif. 2023 Apr 13;56(11):e13480. doi: 10.1111/cpr.13480 (PMC10623941; doi:10.1111/cpr.13480)
Supplement: Supplementary file 1 — Data S1. Supporting information [file CPR-56-e13480-s001.docx]

Supplementary Materials for

**Derivation of new pluripotent stem cells from human extended pluripotent stem cells with formative features and trophectoderm potential**

Pinmou Zhu^1 †^, Bohang Zhang^1†^, Ruiqi Sun^1†^, Jiachen Wang^1†^, Zhaode Liu^1^, Xiaorui Liu^1^, Min Yan^1^, Yiqiang Cui^1*^, Jiahao Sha^2*^, Yan Yuan^1*^

^1^State Key Laboratory of Reproductive Medicine, Nanjing Medical University; Nanjing, 211166, China.

^2^State Key Laboratory of Reproductive Medicine, Women’s Hospital of Nanjing Medical University, Nanjing Maternity and Child Health Care Hospital, Nanjing Medical University; Nanjing, 210029, China.

^†^These authors contributed equally to this work.

^*^Corresponding author. Email: cuiyiqiang@126.com (Y. C.), shajh@njmu.edu.cn (J. S.), yuanyan@njmu.edu.cn (Y. Y.)

This PDF file includes:

Figures S1 to S7

Supplementary Figures and Figure Legends

Table S1

qPCR primers used in this study

Table S2

Summary of chimeric assays of AF9-1 injection at 8-cell embryo stage


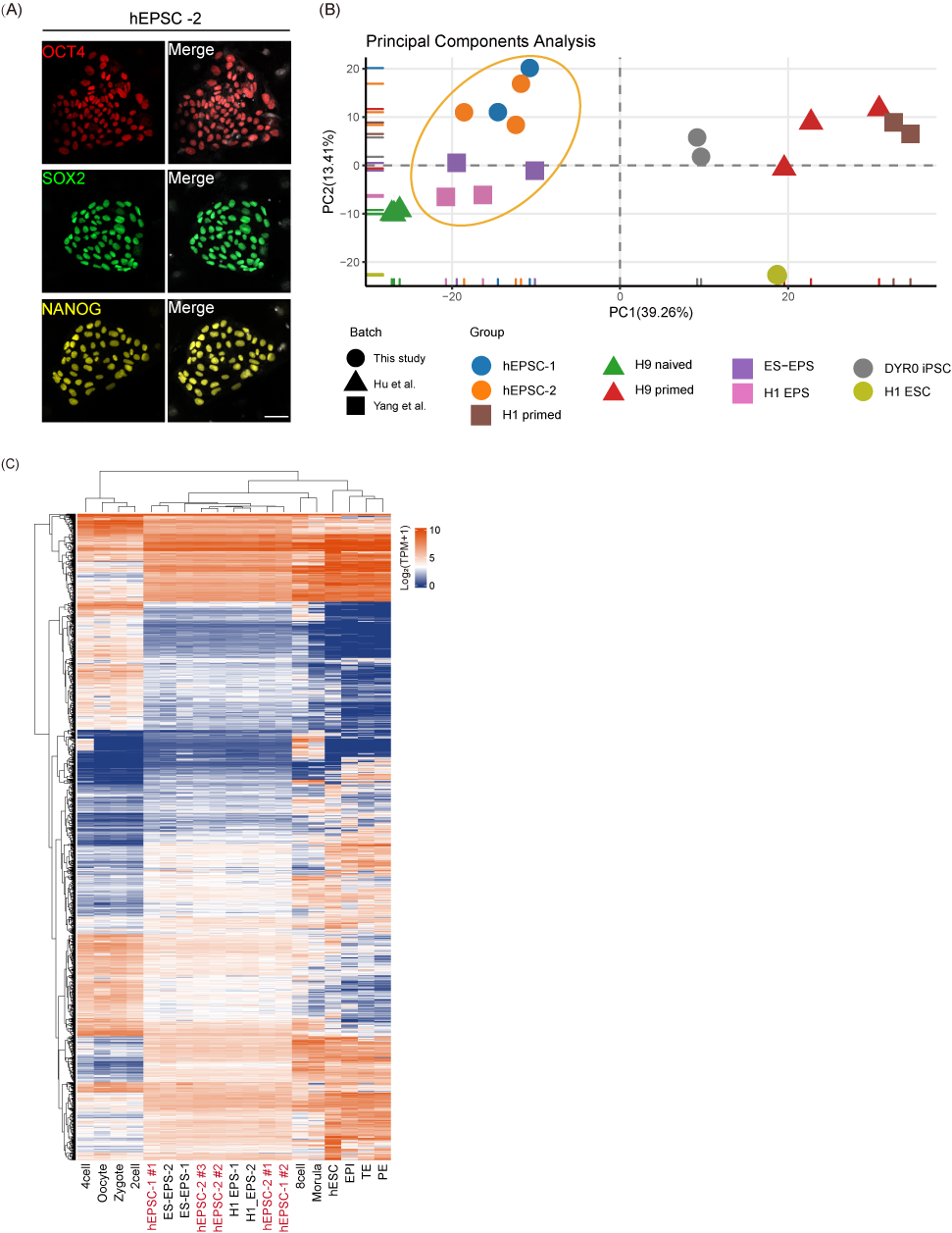
**Figure S1 Establishment of human exended pluripotency stem cells (EPSCs) from primed hPSCs**

1. Representative IF images showing that hEPSC-2 expressed core pluripotency markers (OCT4, SOX2 and NANOG). Scale bar, 50 μm.
2. PCA analysis of RNA-seq data from primed hPSCs (DYR0 iPSC, H1 ESC), hEPSCs (hEPSC-1, -2) and other reported cell types (H9 naïve and primed established by Hu et al. H1 primed, ES-EPS and H1 EPS ­­­established by Yang et al.).
3. Heatmap clustering of indicated EPS cells in (B) and comparison with embryonic cells from preimplantation stages.


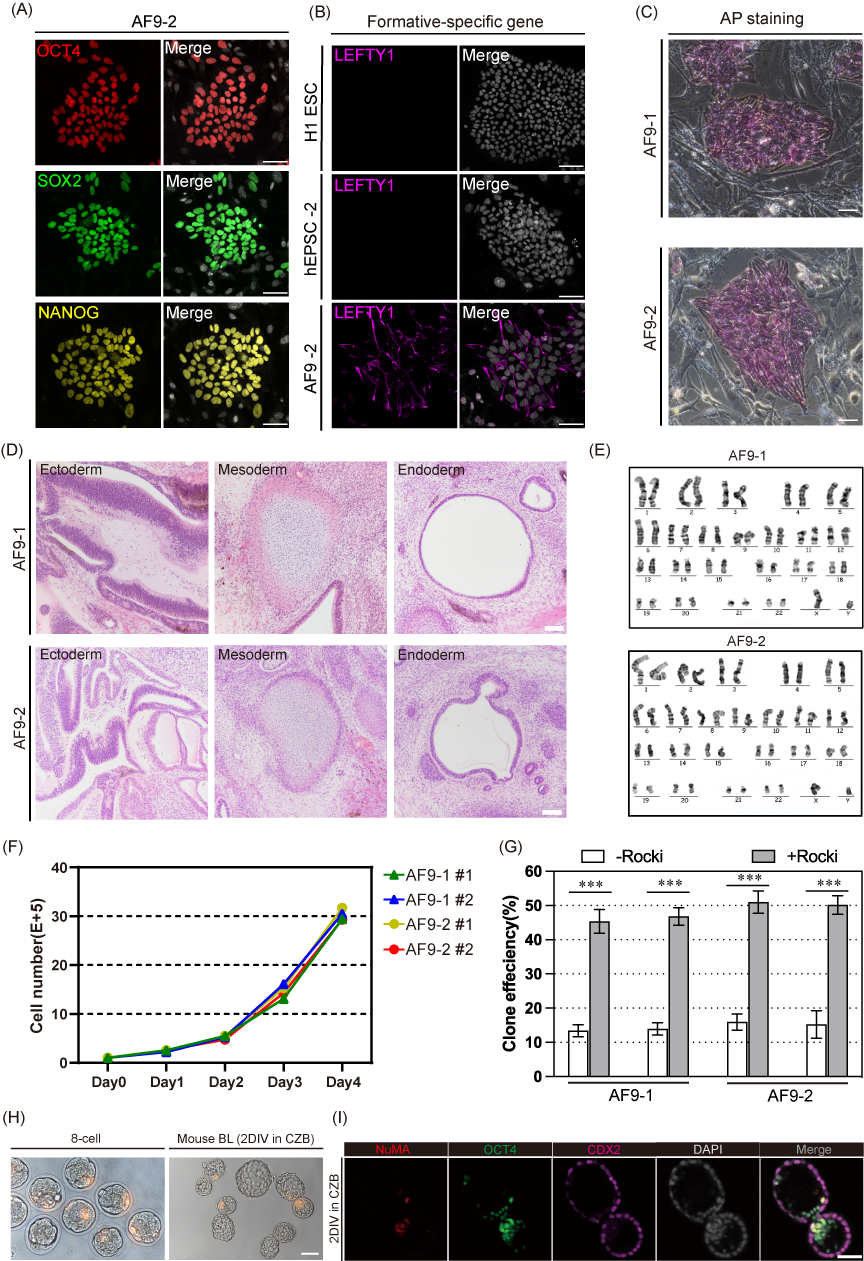
**Figure S2 In vitro deriving for intermediate pluripotent stem cells from human EPSCs**

1. Representative IF images showing that AF9-2 expressed core pluripotency markers (OCT4, SOX2 and NANOG). Scale bar, 50 μm.
2. Representative IF images showing the formative marker LEFTY1 was expressed in formative AF9-2 but not expressed in primed H1 ESCs and hEPSC-2. Scale bar, 50 μm.
3. Alkaline phosphatase (AP) staining of AF9-1 (top) and AF9-2 (bottom). Scale bar, 50 μm.
4. Representative H&E staining images showing that teratomas form by AF9-hPSCs (AF9-1, AF9-2) contained tissues from all three embryonic germ layers. Scale bar, 100 μm.
5. Representative karyotypes of AF9-hPSCs (AF9-1, AF9-2) at passage P15.
6. Growth curves of AF9-hPSCs (AF9-1, AF9-2, passage 10-15).
7. Single-cell clonal efficiencies of AF9-hPSCs (AF9-1, AF9-2) with and without Y27632 treatment (mean±SD; n=3, biological replicates; ***p＜0.001).
8. Representative images showing ICM incorporation of DsRed-labeled AF9-hPSCs in mouse 8-cell embryo. Scale bar, 50 μm.
9. Representative IF images showing DsRed-labeled AF9-hPSCs engrafted mouse ICM and expressed OCT4 following 2 days in vitro (2DIV) embryo culture. Scale bar, 50 μm.


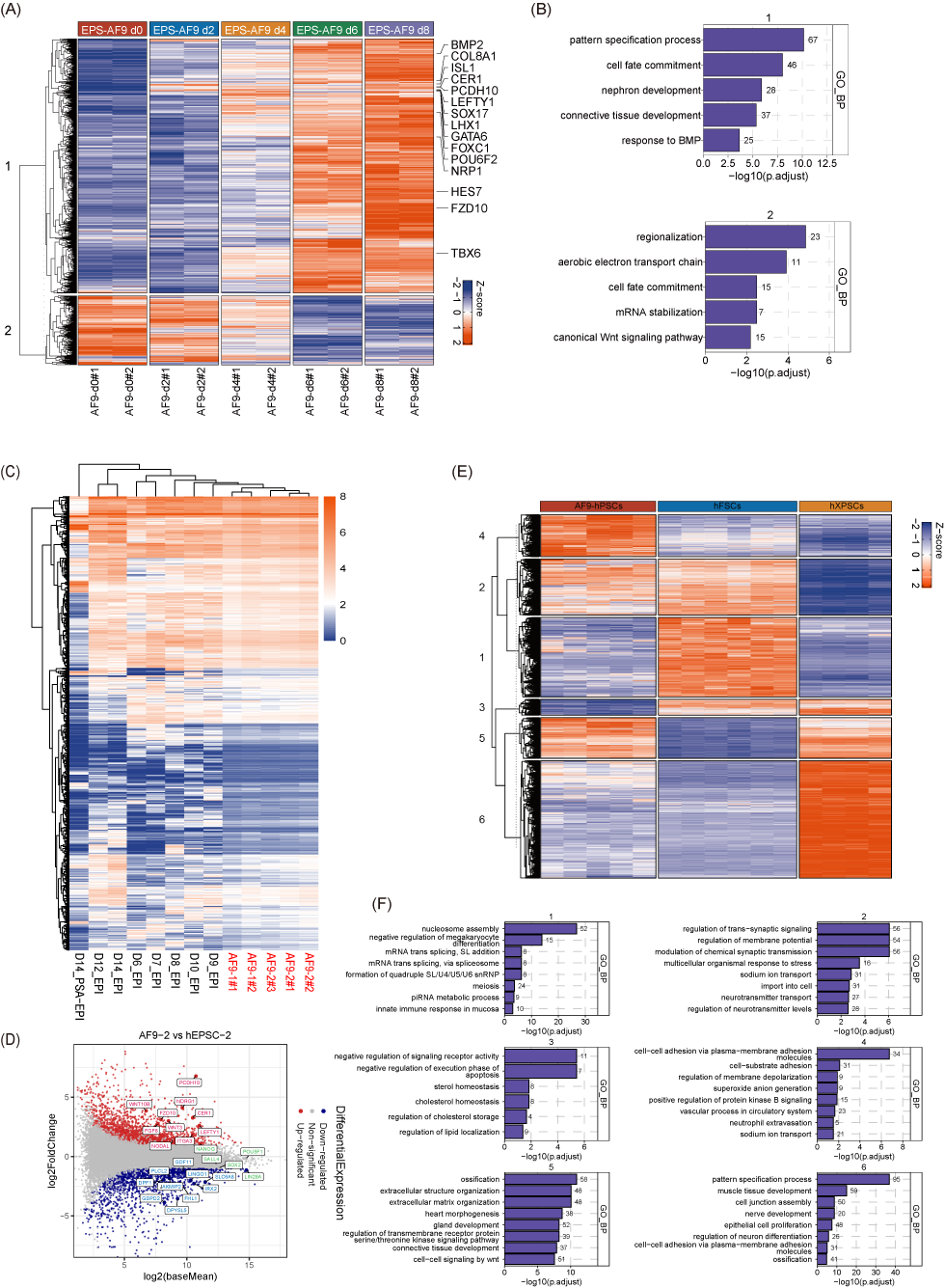
**Figure S3 AF9-hPSCs harbor intermediate pluripotency features**

1. Heatmap clustering of DEGs during AF9 induction from EPS (EPS-AF9 d0, EPS-AF9 d2, EPS-AF9 d4, EPS-AF9 d6, EPS-AF9 d8)
2. Top 5 enriched GO terms of genes specifically expressed in cluster 1 and 2 from (A).
3. Heatmap clustering of indicated AF9-1 and AF9-2 cells and comparison with in vitro culture embryonic epiblast cells, data from Xiang et al (2021).
4. MA plot showing mean FPKM value against fold change per gene in AF9-2 versus hEPSC-2. Gene symbols are shown for selected formative (red), EPS (blue) and pluripotency (green) genes.
5. Heatmap clustering of DEGs in primed AF9-hPSCs (AF9-1, AF9-2) and other reported human formative pluripotent stem cells hFSC, hXPSC.
6. Top 6 enriched GO terms of genes specifically expressed upregulated in AF9-hPSCs, hFSC and hXPSC.


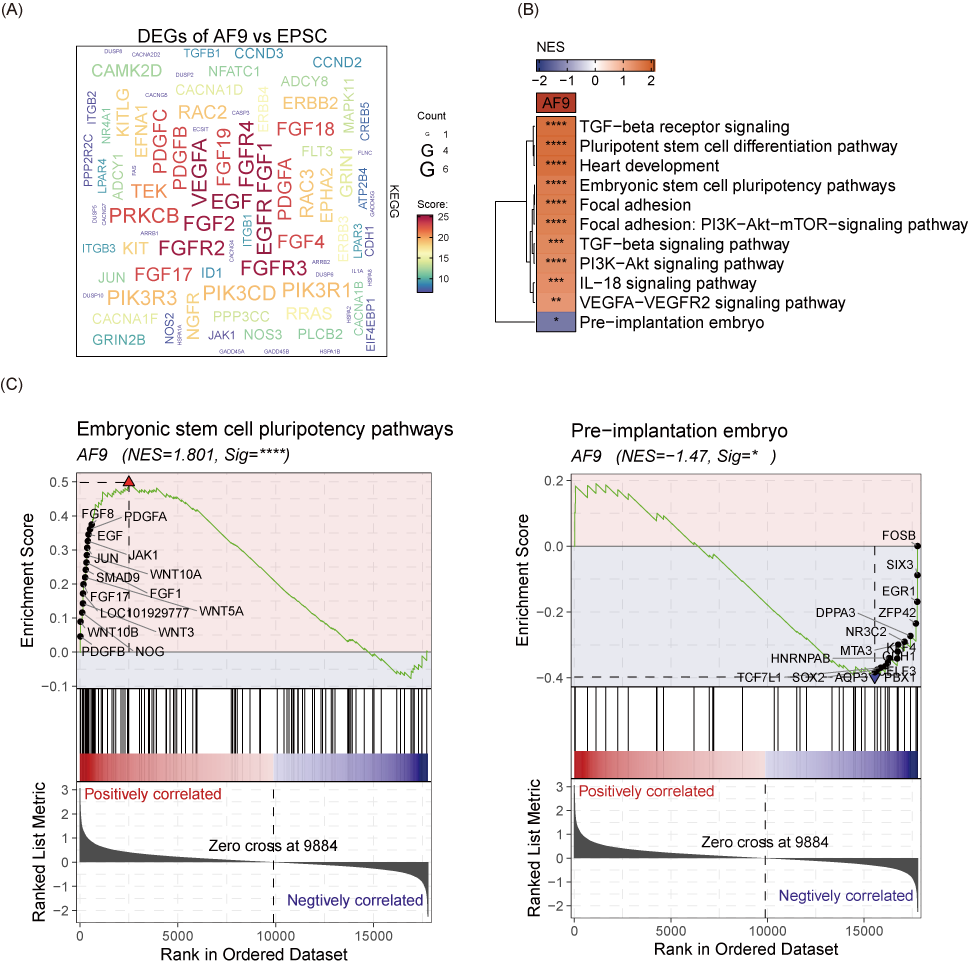
**Figure S4 Analysis of the signaling pathway from hEPSCs to AF9-hPSCs**

1. The word cloud map shows the frequency of enrichment of differentially expressed genes of AF9-hPSCs and hEPSC on the KEGG pathway.
2. Comparison of GSEA pathway analysis based on the WikiPathways database of AF9-hPSCs and hEPSCs. Red indicates positive normalized enrichment score (NES) and blue indicates negative NES (∗p < 0.05, ∗∗p < 0.01, ∗∗∗p < 0.001, ∗∗∗∗p < 0.0001).
3. Enrichment results of GSEA pathway for embryonic stem cell pluripotency pathways and Pre-implantation embryo pathways. The identified genes represent the main up-regulated genes of each pathway in AF9-hPSCs (∗p < 0.05, ∗∗p < 0.01, ∗∗∗p < 0.001, ∗∗∗∗p < 0.0001).


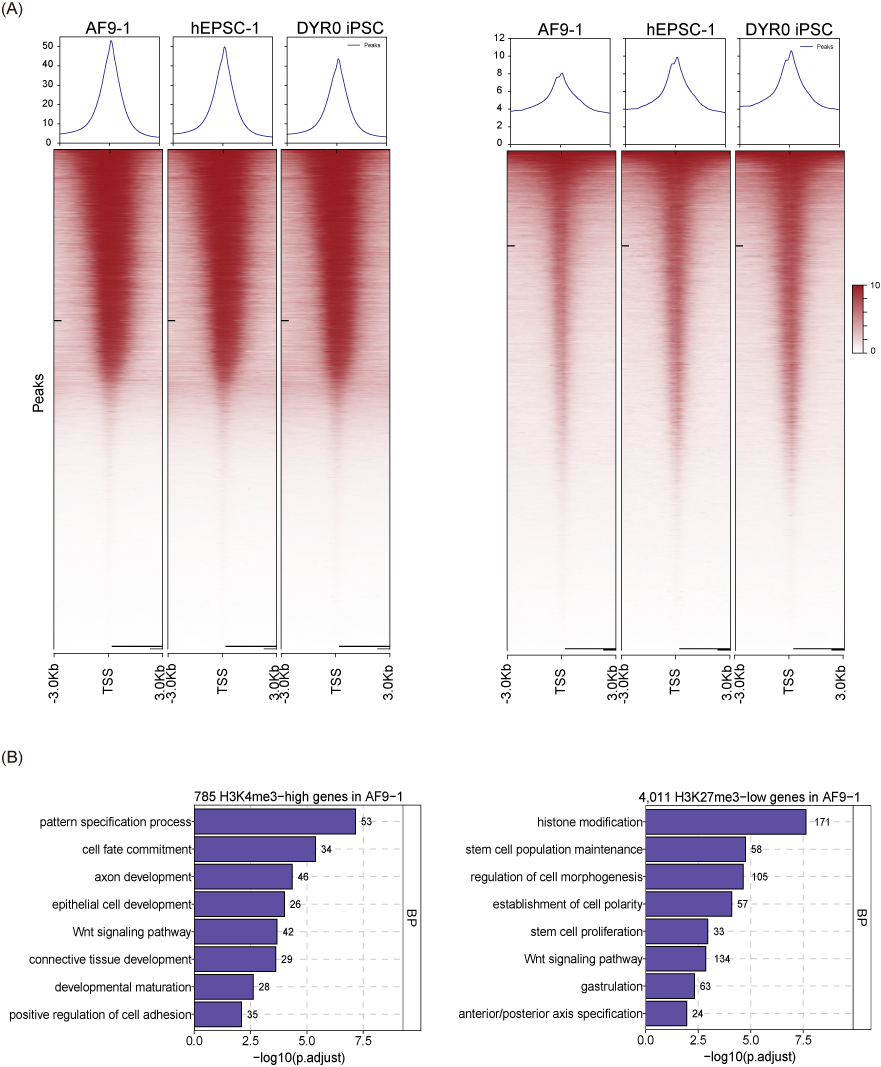
**Figure S5** **Analysis and comparison of CUT&Tag among different cell lines**

1. CUT&Tag signals of H3K4me3 and H3K27me3 for all RefSeq genes AF9-1, hEPSC-1 and DYR0 iPSCs.
2. Top 8 enriched GO items of H3K4me3-high genes and H3K27me3-high genes specifically expressed in AF9-1.


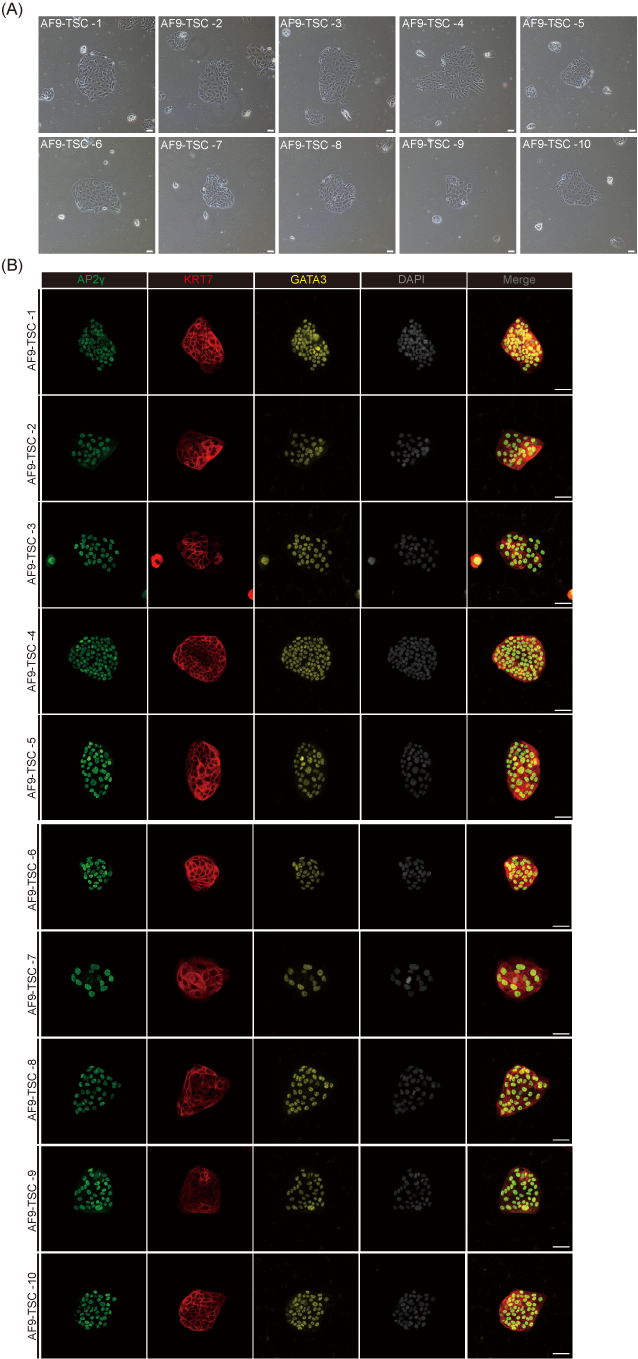


**Figure S6 Derivation of AF9-TSCs from AF9-hPSCs**

(A) Bright images of AF9-TSCs induced from monoclonal AF9-hPSCs. Scale bar, 50 μm.

(B) Immunofluorescence images of AF9-TSCs induced from monoclonal AF9-hPSCs in (A). Scale bar, 50 μm.


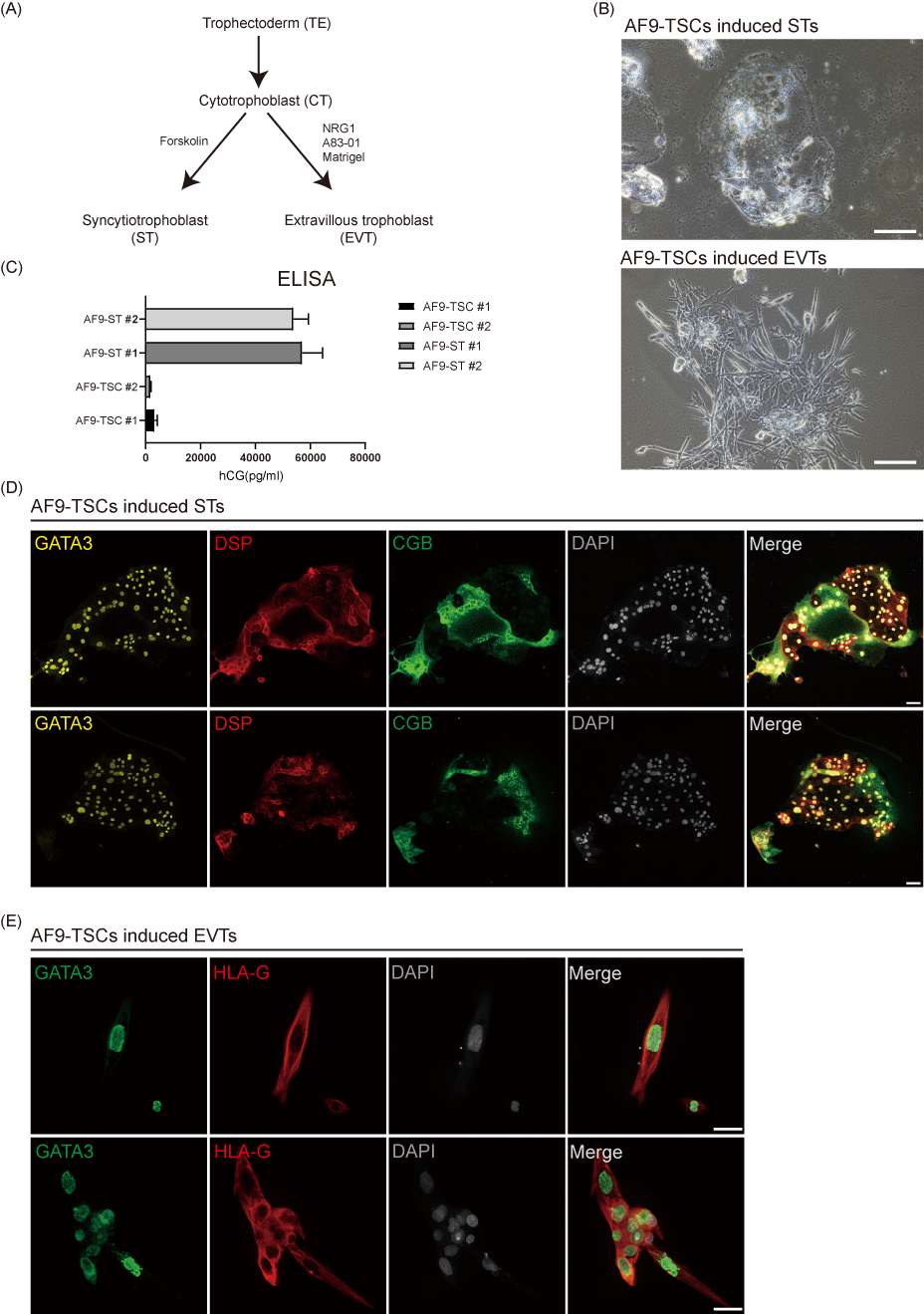


**Figure S7. AF9-TSCs differentiate into STs and EVTs**

(A) Schematic of human trophoblasts. AF9-TSCs were induced into STs in forskolin medium, while induced into EVTs in conditions with NRG1, A83-01 and Matrigel.

(B) Bright image of AF9-STs (TOP) and AF9-EVTs (BOTTOM). Scale bar, 100 μm.

(C) Secretion of human chorionic gonadotropin (hCG) from AF9-TSCs and AF9-STs. Data are presented as mean +SD (n=3).

(D) Immunofluorescence images of AF9-STs. AF9-STs are multinuclear. Scale bars, 50 μm.

(E) Immunofluorescence images of AF9-EVTs. Scale bar, 50 μm.

**Table S1: qPCR primers used in this study.**

| **Genes** | **Forward** | **Reverse** |
| --- | --- | --- |
| GAPDH | GGAGCGAGATCCCTCCAAAAT | GGCTGTTGTCATACTTCTCATGG |
| POU5F1 | CTGTCTCCGTCACCACTCTG | AAACCCTGGCACAAACTCCA |
| ECAD | GGTCTGTCATGGAAGGTGCT | GATGGCGGCATTGTAGGT |
| HHEX | ACGCCCTTTTACATCGAGGAC | CGTGTAGTCGTTCACCGTC |
| FOXA1 | GCAATACTCGCCTTACGGCT | TACACACCTTGGTAGTACGCC |
| FOXA2 | ACCCGGTTTTATCCCTTGAATC | ATACAACCTGCAACCAGACAGG |
| NCAD | TGCACAGATGTGGACAGGAT | CCACAAACATCAGCACAAGG |
| SNAIL1 | GCGAGCTGCAGGACTCTAAT | CGGTGGGGTTGAGGATCT |
| MSGN1 | AGCTCAGGATGAGGACCTTG | CTGGCCTCTCTGGCTGTAGA |
| VIM | TGAGATTGCCACCTACAGGAA | GAGGGAGTGAATCCAGATTAGTTT |
| TBX6 | GAACGGCAGAAACTGTAAGAGG | GTGTGTCTCCGCTCCCATAG |
| ZEB1 | AGCACTTAAGAATTCACAGTGGAG | CATTTCTTACTGCTTATGTGTGAGC |
| PAX6 | GCGGGTGACAAAATAGTTGTCTT | GCCAGGATGTCAAATCTCTCCA |
| SOX1 | GGCCAAGGTAACACTCATCGTA | ACCCTGTGATTTGGGAAGTGAA |
| FOXG1 | GAGCGACGACGTGTTCATC | GCCGTTGTAACTCAAAGTGCTG |
| ZIC1 | CACGCGGGACTTTCTGTTC | TGCCCGTTGACCACGTTAG |
| SOX17 | TTCGTGTGCAAGCCTGAGAT | TAATATACCGCGGAGCTGGC |
| TFAP2C | ATTAAGAGGATGCTGGGCTCTG | CACTGTACTGCACACTCACCTT |
| PRDM1 | AAACCAAAGCATCACGTTGACA | GGATGGATGGTGAGAGAAGCAA |
| NANOS3 | TGGCAAGGGAAGAGCTGAAATC | TTATTGAGGGCTGACTGGATGC |
| DPPA3 | AAGCCCAAAGTCAGTGAGATGA | GCTATAGCCCAACTACCTAATGC |
| PRDM14 | TATCATACTGTGCACTTGGCAGAA | AGCAACTGGGACTACAGGTTTGT |

**Table S2: Summary of chimeric assays of AF9-1 injection at 8-cell embryo stage.**

| **Cell line** | **Host Species** | **Host strains** | **Injected Blastocysts #** | **Culture medium** | **Development Rate %** | **ICM contribution rate %** |
| --- | --- | --- | --- | --- | --- | --- |
| **AF9-1** | **Mouse** | **ICR** | **81** | **CZB** | **85% (69/81)** | **36% (25/69)** |
